# A crossectional investigation of the relationship between complementary health insurance and frequency of dental visits in 15 to 64 years old of Tehran population, Iran, a secondary data analysis (urban HEART-2)

**DOI:** 10.1186/s12913-019-4526-y

**Published:** 2019-09-18

**Authors:** Morteza Rostam Beigi, Ahmad Reza Shamshiri, Mohsen Asadi- Lari, Hossein Hessari, Ahmad Jafari

**Affiliations:** 1 Department of Community Oral Health, School of Dentistry, Tehran University of Medical Sciences, Tehran, Iran; 2 Research Center for Caries Prevention, Dentistry Research Institute, Tehran University of Medical Sciences, Tehran, Iran; 3 Oncopathology Research Center, Iran University of Medical Sciences, Tehran, Iran

**Keywords:** Dental insurance, Dental visit, Dental pain

## Abstract

**Background:**

This study aimed to investigate the relationship between complementary health insurance and frequency of dental visits.

**Methods:**

The present study was performed using the Urban Health Equity Assessment and Response Tool (Urban HEART). A cross-sectional study was conducted in Tehran (Iran) to assess inequalities in health status among different socioeconomic and ethnic groups, genders, geographical areas, and social determinants of health. Out of 20,320 records retrieved from the original study with dental information, 17,252 had both dental insurance and dental visit information. Complementary health insurance as the main independent variable had three categories (i.e., basic insurance, with complementary medical coverage, and with dental coverage). The frequency of dental visits during the last year as a dependent variable had also three categories (i.e., no visit, one, and two, or more dental visits in the last year). In this study, in addition to investigating the relationship between complementary health insurance and frequency of dental visits, potential covariates that may affect the mentioned relationship were evaluated in the regression model. Statistical analyses included simple and multiple multinomial logistic regression considering the sampling method and sampling weights.

**Results:**

The meanage of 17,252 participants (Tehran citizens) was 39.36 years; 49.4%were women, 86.0%hadonly basicinsurance, 7.2% had complementary medical insurance, and 6.8% had complementary dental insurance. Of all subjects, 43.8% reported no dental visit, 26.1% reported one, and 30.1% reportedtwoor more dental visits during the lastyear. The frequency of dental visits was lower in people who had basic insurance than others such that that odds ratio (OR) was 0.73 (*p*-value < 0.001) for one visit and 0.68 (p-value< 0.001) for two or more visits in the last year. The frequency of dental visits was also positively associated with dental brushing, toothpaste use, high educational level, being married, having more than 20 teeth, and having dental pain.

**Conclusion:**

Having dental insurance increases the frequency of dental visits but the association between dental insurance and dental visits was independently influenced by other predictors.

## Background

Dental caries is the most common non-communicable disease in the world [1]. Although regular dental visitsare important indecreasingthe frequency ofdecay-missing-filled index (DMFT) index [2],there are some barriers in this regard. According to thefindings of some previous studies,the high cost of dental servicesis the most common reason for the low frequency of dental visits [3, 4]. Financial status and level of income affect the frequency of dental visits in both developed and developing countries [5–7]. In low and middle-income countries, inequities in health care utilization and out-of-pocket expensesare common; thus, the national health care systems try to facilitate benefiting from dental care in several ways such as insurancedevelopment [8]. In this connection, the World Health Organization (WHO) is one ofthe pioneers of improving health insurance [9]. Individual and social factors can influence the percentage of coverage and quality of health insurance. It has been evidenced that factors such as personal and national income [10], health insurance literacy [11],and socioeconomic status [12] can affect the insurance coverage percentage.

Insurance coverage varies from country to country. For instance,in 2008, 46 million of the United States population were not covered by any kind of health insurance [13]. In addition, in the USA the number of children without dental insurance was 2.6 times more than the number of children without medical insurance [14]. In Turkey, in 2006, although the majority of Turkish people (85%) were covered by some health service program, some of them did not have any insurance coverage.

Insurance coverage is important because it can affect health behaviors. Based on the National Survey of U.S. Children’s Health, Lewis et al. announced that less than half of children without dental insurance received preventive dental care visit in the previous year – i.e., in 2007 [14]. This result is in contrast with results of Yu et al., who reported that dental insurance has a significant role in children’s dental services utilization such as preventive dental visits [15]. However, insurance is not the sole factor to assess the quality of dental care in a society such that other factors such as oral health behaviors, age, sex, and level of education can also affect the oral health status [16].

Iran, having an oil-dependent economy, is among the lower-middle-income group countries [17]. The results of a national survey in 2009 revealed that the share of dental care was 15.5% of the total health costs of Iranian households [18]. A cross-sectional study in Iran investigated the relationship between dental insurance and the type of service received by Iranian dentate adults in 2011.Results showed that more than half of Tehranian citizens with dental insurance had a dental visit within the past 12 months [12].

In 2007, there were 71,330,916 health insurance booklets in Iran that exceeded the total population of the country (i.e., 70,495,000),because some people had two types of insurance while someothers did not have any type ofinsurance coverage [19].

There are four large social insurance funds in Iran. Under the supervision of the Ministry of Cooperation, Labor, and Social Welfare, these funds are responsible for defining packages of basic health benefits and annual tariff adjustments for public and private sectors [18]. The most important insurance companies in Iran are Medical Health Care Services (MSIF) and Social Security Organization (SSO). MSIF is a governmental corporation that covers approximately 35 million people (i.e., government employees, rural households, students, lawyers, physicians and any non-insured volunteers) and the SSO is a non-governmental company with 28 million coverage subjects including formal workers and their families. There are also smaller insurance companies like Armed Forces medical services insurance [18]. MSIF and SSO cover the following basic dental cares for free: Dental visiting (including oral hygiene instruction,examination, and diagnosis, prescribing drugs and radiology, and requesting laboratory tests) tooth extraction, fissure sealant, fluoride therapy, and tooth scaling, and root planing.

Applicants of advanced dental services should buy complementary dental insurance or pay out of pocket [18]. In addition to the percentage of people with health insurance coverage, quality of insurance coverage is an important factor; for example, insurance with and without dental coverage has different effects. Results of a cohort study showed thatroutine dental prophylaxis in people with dental coverage insurance was 52% more than patients without dental coverage [20] Muddassir Siddiqui (2013–2014), through a “Dental Health Screening Program Report” based on the different types of insurance(i.e., Private, Family Health, and First Nation and Inuit Branch), reported that students with dental coverage had better oral health [21].

Although there are a large number of related articles evaluating the relationship between generalhealth insurance and related outcomes, studieson the relationship of dental insurance and oral and dental health determinants are limited especially in Iran as a developing country. Thus,the present study was conducted to investigate the relationship between types of complementary health insurance and the frequency of dental visits as a determinant of oral health utilization in 15–64 years old population of Tehran as independent variables.

## Methods

Following the first round of Urban Health Equity Assessment and Response Tool project (Urban HEART) in 2008, the second round (Urban HEART-2) was conducted on November 2011, within the main framework of WHO Center for Health Development (Kobe Center) to track the changes over time. The present study is a secondary data analysis ofthe second round of Urban HEART-2 study. The main study aimed to measure inequalities in socio-economic determinants and health status in Tehran [22]. Data analysis of the present study was performed from December 2016 to April 2017.Ethics approval was obtained from the Research and Planning Center of Tehran Municipality in 2011 and Ethics Committees ofIran University of Medical Sciences. Although in Urban HEART-2 survey data gathering was conducted via a questionnaire, keeping the name of subjects, images, audiovisual recordings or videos relating to an individual person in private, in the first page of the questionnaire participants over the age of eighteen provided an informed written consent for participation in the project and publication of results [16, 23].

Tehran as the capital and the largest city of Iranis the 23rd most populated city in the world. The area of this metropolis is about 730 km^2^ such that it makes up about 16% of the total population of the country [24]. Tehran is divided into 22 municipal districts and 370 neighborhoods. Socioeconomic status, lifestyle characteristics, and other health determinants arediverse in different districts of the city [24].

The Ethics Committee of TehranUniversity of Medical Sciences approved this study. The participants signed informed consent forms prior to participationin thestudy [16].

Since the age and sex distribution was not proportionateto the population of Tehran, we used the analysis of the complex sample of SPSS softwareto ensure the generalize ability of the results to the Tehran population. To define the complex sample plan, we used 22 districts and 368 neighborhoods as the first and second sampling strata, respectively. Due to security issues, six neighborhoods were not available and multi-stage random sampling was performed forthe remaining 368 neighborhoods [22]. We used blocks as a cluster. The weighting of each respondent was calculated and applied totheage and sex categories of each district. Weighting was based on the national census in 2011 [25].

The original data were collected using three sets of questionnaires collecting the information of 34,000 households (118,000 individuals). Inclusion criteria were being Tehran’s citizen, the ability to understand the questionnaires, and ability to answer the questions. All of 22 districts of the municipality and 368 neighborhoods of Tehran were considered as sample size. GIS maps and software used to select households randomly. Five two-day training workshops of 1240 interviewers were held to train them on how to communicate with the citizens and encourage them to participate in the survey. In Urban HEART-2 study, there were three types of questionnaires; type 1 questionnaires were completed by all selected households in the blocks and the type2 ones (involving oral health questions) were completed by selected individual in each household. The selected individual was the available person who had the most information about the oral health status of household.

More than 24,400 participants responded to the oral health questionnaire consisting of the utilization of dental services, oral health behaviors (using toothbrush and toothpaste), and the number of teeth. According to the purpose of the study, which was assessment of the relationship of dental insurance and utilization of dental services (number of dental visits in thelast year), data cleaning was performed based on two main variables: complementary health insurance and number of dental visits in the last year.

After removing records with missing and invalid data from 24,400 initial oral health-related datasets of urban HEART-2 in Tehran, 17,252 subjects remained for data analyses (response rate = 70.7).

In the present study, sociodemographic characteristics (i.e., gender, age, marital status and education), complementary health insurance, oral health behaviors (i.e., toothbrush and toothpaste usage), having functional dentition (i.e., participants with more than 20 teeth in their oral cavity were considered to have a functional dentition), and having dental pain were considered as demographic and independent variables. On the other hand, the number of dental visits in the last year (as a dental service utilization index) was considered as the dependent variable.

Dental visit as a dependent variable was defined as a categorical variable with three categories (i.e., no dental visit, one, and two or more dental visits in the last year). Complementary health insurance as the main independent variable was defined as a categorical variable with three categories (i.e., basic insurance, with complementary medical coverage and with dental coverage). At the beginning of the statistical analysis, variables that may have been associated with dependent variables were tested in a simple multinomial logistic regression and all comparisons with *P* ≤ 0.20 were selected for multiple regression analysis. These variables are as follows: Education, Marital status, Behavior (Toothpaste usage and Toothbrushing), Number of teeth, and Dental pain. *P*-values less than 0.05 were deemed statistically significant in multiple regression analysis [26]. We checked the probable interactions between tooth brushing and toothpaste usage as well.

## Results

The study subjects consisted of 17,252 people from citizens of Tehran. The sociodemographic characteristics, oral health behavior, and dental pain of the study population are presented in Table 1. Population age range was between 15 and 64 years, with the mean age being 39.36 years; 49.4% (7858 people) were women and 64.9% (*n* = 12,358) were married; 34.2% (*n* = 5230) had college education. Of the total subjects, 86.0% (*n* = 14,709) were with no complementary insurance while 7.2% (*n* = 1298) had only complementary medical insurance without dental insurance and 6.8% (*n* = 1245) had complementary dental insurance as well. Prevalence of no dental visit in last year was 43.8% (*n* = 7572) while 26.1% (*n* = 4462) visited a dentist only once and 30.1% (*n* = 5218) visited a dentist twice or more during this time. Tooth brushing and toothpaste usage were high among subjects as 92% of them announced that they brush their teeth every day (one or more times) and use toothpaste regularly. Prevalence of having functional dentition (more than 20 teeth) was 91.8% (*n* = 14,010) and prevalence of having dental pain in the last year was 27.2% (*n* = 4702).

**Table 1:** Sociodemographic characteristics, oral health behaviors and dental pain in 15–64-year-olds

Variables	N	Percentage^a^
Demographic		
Gender		
Male	7858	49.4%
Female	9394	50.6%
Education		
Under high-school diploma	6173	32.6%
High-school diploma	5796	33.1%
College education	5230	34.2%
Marital status		
Married	12,358	64.9%
Separated or divorced	967	4.8%
Single	3734	30.3%
Dental insurance		
Basic insurance	14,709	86.0%
With complementary medical coverage	1298	7.2%
With dental coverage	1245	6.8%
Behavior		
Frequency of dental visits		
No visit	7572	43.8%
One visit	4462	26.1%
Two or more visits	5218	30.1%
Toothpaste usage		
Regularly	15,709	92.3%
Not regularly	1427	7.7%
Tooth brushing		
No	1447	8.2%
Once or more	15,805	91.8%
Number of teeth		
Less than 20	1570	8.2%
More than 20	14,010	91.8%
Dental pain		
No	12,549	72.8%
Yes	4702	27.2%

^a^Considering sampling weights (age and gender by districts)

Distribution of frequency of dental visit as the main dependent variable of our study among predictor variable is shown in Table 2.

**Table 2:** Frequency distribution of dental visits according to the predictor variables

Variables	Frequency of dental visits		
	No visit n (%)	Once ayear n (%)	Twice or more peryear n (%)
Demographic			
Gender			
Male	3475 (44.2)	2040 (26)	2343 (29.8)
Female	4097 (43.6)	2422 (25.8)	2875 (30.6)
Education			
Under high-school diploma	3103 (50.3)	1420 (23)	1650 (26.7)
High-school diploma	2488 (42.9)	1546 (26.7)	1762 (30.4)
College education	1964 (37.6)	1481 (28.3)	1785 (34.1)
Marital status			
Married	5243 (42.4)	3207 (26)	3908 (31.6)
Separated or divorced	487 (50.4)	214 (22.1)	266 (27.5)
Single	1752 (46.9)	988 (26.5)	994 (26.6)
Dental insurance			
Basic insurance	6606 (44.9)	3770 (25.6)	4333 (29.5)
With complementary medical coverage	498 (38.4)	357 (27.5)	443 (34.1)
With dental coverage	468 (37.6)	335 (26.9)	442 (35.5)
Behavior			
Toothpaste usage			
Not regularly	854 (59.8)	244 (17.1)	329 (23.1)
Regularly	6674 (42.5)	4191 (26.7)	4844 (30.8)
Tooth brushing			
No	875 (60.5)	234 (16.2)	338 (23.4)
Once or more	6697 (42.4)	4228 (26.8)	4880 (30.9)
Number of teeth			
Less than 20	798 (50.8)	291 (18.5)	481 (30.6)
More than 20	5982 (42.7)	3765 (26.9)	4263 (30.4)
Dental pain			
Pain	649 (27.6)	630 (26.8)	1075 (45.7)
No pain	6923 (46.5)	3831 (25.7)	4143 (27.8)

In order to explore the relationship between the demographic and other independent variables with the frequency of dental visit as the main dependent variable, first, we performed simple multinomial logistic regression analysis (Table 3). The variables for the multiple regression analysis model were selected based on comparisons with *P* ≤ 0.20 and *P* values less than 0.05 were deemed statistically significant [26]. As shown in Table 3, there was no significant relationship between frequency of dental visit with gender and age and thus they were not entered the multiple regression models.

**Table 3:** Relationship between frequency of dental visits and independent variables in Tehran citizens: bivariate data analyses

	Once a year vs. no visit			2 and more visits vs. no visit		
	OR^a^	95%CI	*P*-value	OR^a^	95%CI	*P*-value
Demographic						
Gender						
Male	1.00	0.91–1.11	0.85	0.93	0.84–1.02	0.12
Female	1.00			1.00		
Education						
Under high-school diploma	0.66	0.58–0.75	< 0.001	0.60	0.54–0.68	< 0.001
High-school diploma	0.86	0.76–0.98	0.02	0.80	0.71–0.89	< 0.001
College education	1.00			1.00		
Marital status						
Married	1.17	1.04–1.32	0.01	1.28	1.14–1.44	< 0.001
Separated or divorced	0.84	0.68–1.06	0.14	1.00	0.79–1.26	0.98
Single	1.00			1.00		
Dental insurance						
Basic insurance	0.73	0.60–0.88	< 0.001	0.68	0.56–0.82	< 0.001
With complementary medical coverage	0.82	0.63–1.07	0.14	0.87	0.66–1.16	0.34
With dental coverage	1.00			1.00		
Behavior						
Toothpaste usage						
Not regularly	0.48	0.40–0.58	< 0.001	0.60	0.5–0.73	< 0.001
Regularly	1.00			1.00		
Tooth brushing						
No	0.44	0.36–0.53	< 0.001	0.57	0.49–0.68	< 0.001
Once or more	1.00			1.00		
Number of teeth						
Less than 20	0.61	0.51–0.73	< 0.001	0.78	0.67–0.9	< 0.001
More than 20	1.00			1.00		
Dental pain						
Pain	1.85	1.59–2.14	< 0.001	3.07	2.66–3.55	< 0.001
No pain	1.00			1.00		

^a^Unadjusted odds ratios were calculated by simple multinomial logistic regression considering complex sample analysis

Results of multivariable data analysis are shown in Table 4. The dental visit was significantly more frequent in college-educated people in comparison with people with under highschool diploma and people with a high-school diploma.

**Table 4:** Relationship between frequency of dental visits and independent variables in Tehran citizens (multivariable data analysis)

	Once a year vs. no visit			2 and more visits vs. no visit		
	OR*	95%CI	*P*-value	OR^a^	95%CI	*P*-value
Demographic						
Education						
Under high-school diploma	0.70	0.61–0.8	< 0.001	0.60	0.52–0.69	< 0.001
High-school diploma	0.84	0.74–0.96	0.01	0.76	0.67–0.86	< 0.001
College education	1.00			1.00		
Marital status						
Married	1.29	1.13–1.47	< 0.001	1.39	1.22–1.58	< 0.001
Separated or divorced	1.04	0.81–1.32	0.78	1.15	0.87–1.51	0.32
Single	1.00			1.00		
Dental insurance						
Basic insurance	0.78	0.64–0.95	0.01	0.79	0.65–0.96	0.02
With complementary medical coverage	0.90	0.69–1.17	0.42	1.01	0.77–1.32	0.96
With dental coverage	1.00			1.00		
Behavior						
Toothpaste usage						
Not regularly	0.70	0.57–0.88	< 0.001	0.86	0.67–1.1	0.24
Regularly	1.00			1.00		
Tooth brushing						
No	0.57	0.46–0.7	< 0.001	0.60	0.49–0.75	< 0.001
Onceormore	1.00			1.00		
Number of teeth						
Less than 20	0.69	0.57–0.83	< 0.001	0.83	0.71–0.98	0.03
More than 20	1.00			1.00		
Dental pain						
Pain	1.87	1.6–2.19	< 0.001	3.23	2.77–3.78	< 0.001
No pain	1.00	0.64–0.95		1.00	0.65–0.96	

^a^Adjusted odds ratios were calculated by multiple multinomial logistic regressions considering complex sample analysis

The frequency of dental visits in under high school diploma educated people (OR = 0.66, CI: 0.58–0.75; *p*-value < 0.001) and high school diploma degree (OR = 0.86, CI: 0.76–0.98; *p*-value = 0.02) was lower than that of university graduates. Also, the frequency of two or more dental visits in the last year in people with under high school diploma (OR = 0.60, CI: 0.54–0.68;p-value< 0.001) and those with high school diploma degree (OR = 0.80, CI: 0.71–0.89; p-value< 0.001) was lower than university graduates.

Frequency of dental visit was higher among married people as a dental visit for once per year (OR = 1.17, CI: 1.04–1.32; *p*-value = 0.01) and 2 or more per year (OR = 1.28, CI: 1.14–1.44, *p*-value< 0.001) was higher among married people in comparison to others. Data analysis showed that dental visit was lower in people without dental insurance in the last year.

The results of unadjusted complex samples logistic regression showed that people who had basic insurance in comparison with people with dental complementary insurance, visited a dentist less frequently for both states of dental visit: one visit (OR = 0.73,CI: 0.60–88; *p*-value< 0.001) andtwice or more visits (OR = 0.68,CI: 0.56–0.82; *p*-value< 0.0011) during the last year.

According to the results, there was no significant difference between numbers of the dental visit in people with medical complementary insurance compared to those with basic insurance in once (*p*-value = 0.14) and twice or more dental visit (*p*-value = 0.34).

The dental visit was significantly higher in people with better oral health behavior (once a day and twice or more a day brushing, and regular toothpaste usage). The frequency of once per year dental visit (OR = 0.44, CI: 0.36–0.53; *p*-value< 0.001) and twice or more per year (OR = 0.57, CI: 0.49–0.68; p-value = 0.00) was higher in people with once or more time daily brushing. Also, the regular toothpaste users visit a dentist once per year and higher than those who were not regular toothpaste users (OR = 0.48, CI: 0.40–0.58; *p*-value < 0.001) or have twice or more visits per year (OR = 0.60, CI: 0.50–0.73; p-value < 0.001).

The dental visit was significantly greater in people with functional dentition (more than 20 teeth). During the last year, people with less than 20 teeth in their oral cavity in comparison to people with more than 20 teeth had less one-time dental visit (OR = 0.61, CI: 0.51–0.73; *p*-value < 0.001) and twice or more dental visits (OR = 0.78, CI: 0.67–0.90; p-value < 0.001). People with dental pain experience visited a dentist more frequently in the last year. People without history of dental pain had lower chances of visiting a dentist in the last year, in both state of dental visit: once in year (OR = 1.85, CI: 1.59–2.14; p-value < 0.001) and twice or more visits per year (OR = 3.07, CI: 2.66–3.55; p-value < 0.001).

No significant interactions were found; so, they were not included in our analysis**.**

## Discussion

The present study is aimed to investigate the relationship between types of complementary health insurance and the frequency of dental visits as a determinant of oral health utilization in 15 to 64 years old of Tehran population as independent variables. The results showed that the frequency of dental visits was significantly more in people with complementary dental insurance, those with regular tooth brushing and toothpaste usage habits, married individuals, having more than 20 teeth, and people with experience of dental pain.

The strong points and limitations of the main study (i.e., Urban HEART-2) were evaluated and secondary data were analyzed:

The present study, despite its large sample size and multidisciplinary and well-designed method, had some limitations:

- In addition to the cross-sectional design of the study, time limitation of data collection (25 days across 22 districts of Tehran) led to enormous pressure on field surveyors. As a result, 8 neighborhoods (out of 374) were omitted from data collection due to non-residential or military blocks;
- Long and detailed questions made some respondents refrain from completing questionnaires.
- The time of the survey was from morning to noon (day time) and thus mainly woman (> 60%) responded to the personality questionnaires. In Urban HEART-2, the same households or blocks that had been involved in Urban HEART − 1 were not followed.

Various strategies have been developed to modify the mentioned limitations that are available in the Urban HEART-2 papers [22].

In the present study, despite the large sample size, missing data posed a limitation. As mentioned, more than 24,400 participants responded to the oral health questionnaire consisting of utilization of dental services, oral health behaviors (using toothbrush and toothpaste), and number of teeth. According to the purpose of the study, which was evaluation of the relationship between dental insurance and utilization of dental services, data cleaning was performed based on two main variables: complementary health insurance and number of dental visits in the last year. After removing records with missing and invalid data, 17,252 subjects remained for data analyses (response rate = 70.7). The high number of missing data could affect the results of the study considered as a limitation.

Other predictors that were not evaluated in our study, such as the reason fordental visit avoidance, should be considered independently to clarify the significant relationship betweendental visits and dental insurance. This can be considered as another limitation of our study and should be addressed in future studies.

Some studies confirmed the positive effectof the dental visit on various components of oral and dental health such as better dental status, tooth loss, and higheroral health-related quality of life [27, 28].

In the present study, there was no significant difference between age and sex in oral health utilization (frequency of dental visits). In agreement with our findings, Blasi et al. (2018) inthe US found that there was no association between dental care utilization and age and gender [29]. In some studies, significant differences were identified between demographic status (age and sex) and dental care utilization in various social groups. Nazliel et al. (2012) announced that there were no gender-based differences in frequency of dental visits in elderly population in Ankara [30].

Our results showed that the frequency of yearly dental visit was higher in people with university education. One study in Brazil showed that the probability of dental service utilization was higher among people with more than 8 years of education [31]. Another study in South Korea revealed that higher level of education wasa ssociated with a higher prevalence of preventive dental visits [32]. Also, in the middle-aged and elderly population of northeast China, the level of education was positively associated with the frequency of dental visits [33]. Elsewhere, a positive relationship has been reported between the frequency of dental visits and educational level [34, 35]. Our results showed that married people had more dental visits during the last year. In agreement with our results, some studies have shown that the frequency of dental visitswas influenced by marital status [36, 37] while some others did notreport such a correlation [38, 39].

Our results showed that people with dental insurance had more dental visits during the last year. Dental insurance has been addressed as a factor with a positive association with the use of dental services [40]. For decades, dental researchers mentioned that there was a relationship between dental insurance and frequency of dental visits. Manski et al. (1987) reported that dental insurance is an essential predictor of dental care utilization [41]. To date, the relationship between dental insurance and frequency of dental visits has been a matter of debate [42].

In a previous study, 82%of the population with dental insurance had dental visits at least twice a year [43] while in our study, 62%of the population with dental insurance visited a dentist at least once during the last year. Most previous studies haveacknowledged the significant relationship of dental insurance with frequency of dental visits but some studies suggest the role of more important factors. One study reportedthat having health insurance did not ensure more frequent dental visits [44].

In our study, oral health behaviors such as toothbrushing and toothpaste usage were evaluated. The results showed that the mentioned two variables had a significant effect on the final model (independently and with no interaction) and consequently both of them were positively correlated withthe frequency of dental visits. The results of some studies were in line with our findings while some others were not. Hill et al. showed that the pattern of dental visits was associated with the frequencyof tooth brushing [45]. In a study in Santiago (Chile), Lopez et al.reported that behavioral factors were independently related to the frequency of dental visits [46].

The results of a Brazilian study showed that there was a positive relationship between functional dentition and frequency of dental visits in the previous 12 months,which confirmed our results [47]. Evaluation of determinants of dental service utilization in a community-dwelling elderly Japanese population showed that the higher number of remaining teeth was a significant predictor of dental care utilization during the last year as a regular dental visit was associated with a higher number of existing teeth [48].

Several studies confirmed the significant relationship between dental pain and dental visit and addressed a strong association between dental visits and dental pain [49, 50].

Given that Iran is considered a low-middle income country and because of a considerable share of dental care from the total health cost of each household (15.5%), dental insurance can increase dental visits. Also, it can change the present treatment-based approach of dental visits to a prevention-focused one, leading to the improved oral health status and reduced costs of dental care.

It is noteworthy that other predictors, such as the reason of dental visit avoidance, were not evaluated in our study and thus should be considered independently to clarify the significant relationship of dental visits and dental insuranceand addressed in future studies.

## Conclusion

In agreement with our study, other studies have demonstrated that dental insurance has a positive association with dental service utilization. Based on the obtained results, individuals with dental insurance were more likely than their uninsured counterparts to visit a dentist; however, this association was independently influenced by other predictors. Development of dental insurance should be considered in Iran and concurrently other factors of dental visits avoidance should be evaluated and eliminated. Finally, the development of dental insurance leads to an increase in the frequency of dental visits. In this regard, further attempts are needed to shift insured people from treatment focused dental visits to a preventive focused one.

## Data Availability

Based on a mutual agreementbetween researchers and Social and Cultural Affairs Deputy of the Municipality of Tehran, the datasets used and/or analyzed during the present study are not publicly available. However, it will be available from the corresponding author for a reasonable request.

## Abbreviation

Urban HEART: Urban Health Equity Assessment and Response Tool
